# Effect of aligners on patients’ oral health-related quality of life and anxiety: a prospective pilot study

**DOI:** 10.1186/s40359-024-01834-2

**Published:** 2024-06-13

**Authors:** Panagiotis Roulias, Georgios Vasoglou, Gerassimos Angelopoulos, Nikolaos Pandis, Iosif Sifakakis

**Affiliations:** 1 Department of Orthodontics, School of Dentistry, National and Kapodistrian University of Athens, Athens, Greece; 2 Department of Orthodontics and Dentofacial Orthopedics, Dental School/Medical Faculty, University of Bern, Bern, Switzerland

**Keywords:** Orthodontic, Aligner, Oral health related quality of life, Anxiety, Questionnaire

## Abstract

**Background:**

This prospective study explored the impact of aligners on the oral health-related quality of life and anxiety of patients during the first month of orthodontic treatment and the first month of the retention phase.

**Methods:**

A total of 23 male and female patients (median age 25 y) treated with clear aligners were included. The OHRQoL questionnaire was used at certain time points during treatment (T1: placement of the first aligner; T2: after one day of use; T3: after seven days; T4: after one month; and T5: after one month in the retention phase). The State-Trait Anxiety Inventory (STAI) was also self-administered to assess state and trait anxiety (Y1 and Y2 subscales, respectively) at the T1, T4 and T5 time points. A population average generalized estimating equations logistic regression model was fit to assess the effect of time on the responses, and the Wald test was used to examine the overall effect of time.

**Results:**

Overall time was a significant predictor for most of the questions. However, time was marginally significant for the OHRQoL questions evaluating oral symptoms such as bad taste/smell, sores, and food accumulation. Tooth discolouration did not differ between time points. The general activity disturbance was significantly lower in the retention phase. Higher depression and anxiety scores were reported at the initial appointment and decreased thereafter.

**Conclusions:**

CAT has a negative impact on quality of life and psychological status during the initial days of treatment. These impairments ameliorate at later treatment stages.

## Contributions to the literature

• There is little evidence on the interaction between patient quality of life and anxiety levels during the first month of the clear aligner treatment and after one month of the retention phase.

• This prospective study aimed to investigate the potential effect of CAT on patient well-being over time.

## Introduction

Patients today are more aware of any postoperative symptoms related to dental operations, which may increase patient anxiety and stress. Over the initial stages of any orthodontic treatment, patients often experience different levels of pain, discomfort, and anxiety, which can compromise daily activities such as eating and sleeping [1, 2]. Greater anxiety levels have been associated with more painful and long-lasting postoperative periods [3].

Anxiety is defined as the cognitive perception of a vague or ambiguous, subjective threat, producing psychological and behavioral changes as well as further physiological responses [4]. Anxiety may affect a person’s social, psychological, and physical status [5, 6] and quality of life (QoL). Several QoL indices have been implemented in clinical research for the evaluation of patient experience and perception regarding the intervention itself and postoperative recovery [7–10]. In the dental field, “Oral Health-Related Quality of Life” (OHRQoL) focuses on quality of life linked to oral health [11–13]. The OHRQoL reflects people’s comfort when eating, sleeping and engaging in social interaction; their self-esteem; and their satisfaction with respect to their oral health. It is the result of an interaction between and among oral health conditions, social and contextual factors, and the rest of the body [11]. The literature presents various tools aimed at quantifying OHRQoL by estimating patient difficulties associated with discomfort, pain, mastication difficulties, speech disturbances and social impairments [12, 14–19].

OHRQoL scores during orthodontic treatment seem to be negatively affected by physical discomfort, pain, functional problems, and psychological issues [20, 21]. Nevertheless, these scores improve substantially with time [20–22]. Clear aligner treatment (CAT) was associated with better OHRQoL during orthodontic treatment than was treatment with fixed appliances [23]. CAT is becoming increasingly popular among patients seeking orthodontic therapy. However, this system has serious limitations regarding the accuracy of expected tooth movements compared to conventional fixed appliance orthodontic treatment [24]. Studies on CAT have shown better patient-reported experiences in oral hygiene, comfort, esthetics, pain, periodontal status, patient chair-time and overall treatment time [24–27]. A recent study demonstrated that the levels of pain, painkiller intake and quality-of-life measures during CAT increased on the first day and decreased at 3 months [23]. Another recent study evaluated OHRQoL and oral hygiene in adolescents during the first year of aligner therapy. They found that OHRQoL was only slightly affected and that oral hygiene at home was intensified [28]. However, the type of appliance used influences the pain and quality of life of patients at the start of orthodontic treatment [29].

The aim of this study was to investigate the effect of CAT on anxiety levels and OHRQoL in late adolescent/adult patients at different time points during the first month of treatment and at the end of the first month of retention. The null hypothesis was that CAT treatment would not affect stress levels or the OHRQoL at these time points.

## Materials and methods

The sample for this study was prospectively recruited from a private practice limited to Orthodontics in Athens, Greece, between January and November 2022. All patients were asked to participate voluntarily in this study by self-answering both the OHRQL and the STAI Y1 and Y2 [Adult State Anxiety Scale (STAI-AD) (S-Anxiety) and Trait Anxiety Scale (T-Anxiety)] questionnaires. Patients who met the following inclusion criteria were eligible to participate in the study: had (a) a Caucasian origin, (b) were older than 16 years old and seeking CAT and had good communication skills in the Greek language, (d) needed treatment for both dental arches, (d) had a dental health component of the Index of Orthodontic Treatment Need IOTN less than 3 [30], and (d) had a CAT treatment plan that included neither attachment placement nor interproximal enamel reduction (IPR) during the first month. The exclusion criteria were (a) inherited or acquired craniofacial deformities; (b) previous orthodontic treatment; and (c) chronic treatment with anti-inflammatory drugs, analgesics and/or anxiolytics. Patients who failed to cooperate with the given instructions during orthodontic treatment or who lost their aligners during the observation time period were excluded from the study. This was a per-protocol analysis in which the duration of the patient recruitment phase was 1 month. All patients were treated by the same clinician with the same aligner system under the same attachment bonding clinical protocol and were given specific instructions concerning aligner maintenance and postoperative clinical complications such as pain. Patients were asked to change their aligner every 7 days. OHRQoL was completed immediately after the first aligner was delivered (T1), after one day (T2), after seven days (T3) and after one month of aligner use (T4), as well as after one month in the retention phase (T5). All the STAI questionnaires were completed at T1, T4 and T5. The questionnaires were distributed to the patients, and specific instructions were given on when to complete them. Patients were instructed to answer all questions from each questionnaire only once. Reminder phone calls were made for all participants at T2-T5. The duration of all CAT treatment plans included in the study was ≤ 3 months. The retention protocol included canine-to-canine fixed retainers and Essix appliances night-time wear.

### OHRQoL questionnaire

The Greek version of the OHRQoL questionnaire, which consists of 16 questions, was used (Appendix). Question 1 (Q1) was scored on a 1–10 scale (1 = not at all; 10 = very much), question 2 (Q2) on a 2-level scale (0 = no; 1 = yes), and questions 3–16 (Q3-Q16) on a 5-level scale: 1 = not at all; 2 = very little; 3 = a little; 4 = quite a lot; 5 = very much. A last question was added regarding the subjective evaluation of tooth discoloration on a 5-point scale.

### STAI questionnaires

The State-Trait Anxiety Inventory (STAI) – form Y consists of a self-assessment index on both state and trait anxiety in adult individuals. The “state” and “trait anxiety” terms were presented by Cattell [31, 32] and further analyzed by Spielberger [33–36]. State anxiety (S-Anxiety) is defined as an organism’s transient emotional condition and is characterized by subjective feelings of tension and apprehension [37]. Trait anxiety (T-Anxiety) is a stable, anxious propensity to perceive people and situations as threatening, thus increasing anxiety. The adult version of the STAI questionnaire (STAI-AD) consists of 40 items distributed into two 20-item scales. The S-Anxiety Scale, also known as the STAI Form Y-1, evaluates a person’s feelings “right now, at this moment”, while the T-Anxiety Scale, known as the STAI Form Y-2, assesses a person’s general feelings [38]. All the subscales (STAI-Y1 and STAI-Y2) have 20 items scored on a Likert-type scale, with four response options (0 to 3). The expected average completion time is 10 min. The questionnaire version has been used previously in various Greek samples and has been shown to have good internal consistency [36]. Cronbach’s alpha was 0.93 for the State subscale and 0.92 for the Trait subscale in the initial validation study in the Greek population [38]. License to reproduce the STAI-AD was obtained for the aim of this study.

### Statistical analysis

Descriptive statistics were calculated per question for the OHRQoL questionnaire. Q3-8 were combined since they evaluate difficulties speaking, swallowing, opening the mouth and eating (oral dysfunction). In a similar fashion, Q9-11 and Q12-16 were merged, and a summary score was calculated per question group by adding the individual scores across the corresponding questions. For Q1, Q3-8, Q9-12, and Q12-16, a population average Gaussian generalized estimating equation (GEE) regression model with robust standard errors and nonparametric bootstrapping (500 repetitions) was fit to assess the effect of time on the summed responses of the questionnaires. The Wald test was used to test the overall effect of time.

For Q2, a population average GEE logistic regression model with robust standard errors was fit to assess the effect of time on the responses of the questionnaires. The Wald test was used to test the overall effect of time, and the predicted probabilities for response 1 were calculated. The 5 levels of Q17 were reduced to 3 levels because the last 3 levels had a very low number of events. A population average ordinal GEE regression model with robust standard errors. The Wald test was used to test the overall effect of time.

For both the STAI-Y1 and STAI-Y2, a summary score was calculated by adding the individual responses across the corresponding items, and a Gaussian GEE model with robust standard errors and nonparametric bootstrapping (for 500 repetitions) was fit to examine the effect of time on the summed response. The overall effect of time was examined using the Wald test. Predicted effects over time were plotted for each dependent variable. All analyses were conducted using Stata 17 (Stata Corp., TX, USA).

## Results

A total of 23 patients (12 females, 11 males) were included in the study (Table 1). The descriptive statistics for each variable and time point are shown in Table 2.

**Table 1:** Descriptive statistics for patient demographic data

**Age,** median (IQR) (years)	25 (23,42)
**Education**	
Secondary	9 (39%)
Tertiary	14 (61%)
**Occupation**	
Missing	2 (8.7%)
Private practice	13 (57%)
State employee	1 (4.3%)
Unemployed	7 (30%)

**Table 2:** Descriptive statistics for the recorded variables at each time point [mean (SD); n(%)]

**Variable**			**Time**		
	**T1**	**T2**	**T3**	**T4**	**T5**
**OHRQL: Question 1**					
1	4 (17%)	4 (17%)	6 (26%)	12 (52%)	8 (35%)
2	9 (39%)	4 (17%)	9 (39%)	7 (30%)	3 (13%)
3	2 (7%)	4 (17%)	3 (13%)	2 (7%)	3 (13%)
4	2 (7%)	2 (7%)	2 (7%)	0	1 (4%)
5	3 (13%)	5 (22%)	0	0	1 (4%)
6	1 (4%)	0	1 (4%)	0	1 (4%)
7	0	2 (7%)	2 (7%)	2 (7%)	0
8	1 (4%)	0	0	0	0
9	1 (4%)	2 (7%)	0	0	0
10	0	0	0	0	0
Missing	0	0	0	0	6
**OHRQL: Question 2**	2 (8.7%)	7 (30%)	6 (26%)	3 (13%)	2 (12%)
Missing	0	0	0	0	6
**OHRQL: Questions 3-8**	9.3 (2.6)	10.5 (3.4)	8.9 (3.2)	9.1 (3.7)	5.9 (4.2)
**OHRQL: Questions 9-11**	4 (2)	27 (6)	48 (10)	70 (15)	86 (18)
**OHRQL: Questions 12-16**	5.87 (1.29)	6.78 (2.58)	7.04 (3.04)	6.48 (2.13)	4.91 (3.63)
**OHRQL: Question 17**					
1	19 (83%)	17 (74%)	14 (61%)	13 (57%)	10 (59%)
2	3 (13%)	3 (13%)	7 (30%)	6 (26%)	4 (24%)
3	1 (4.3%)	2 (8.7%)	1 (4.3%)	2 (8.7%)	2 (12%)
4	0 (0%)	0 (0%)	0 (0%)	2 (8.7%)	1 (5.9%)
5	0 (0%)	1 (4.3%)	1 (4.3%)	0 (0%)	0 (0%)
Missing	0	0	0	0	6

### OHRQoL questionnaire

**Fig. 1:**
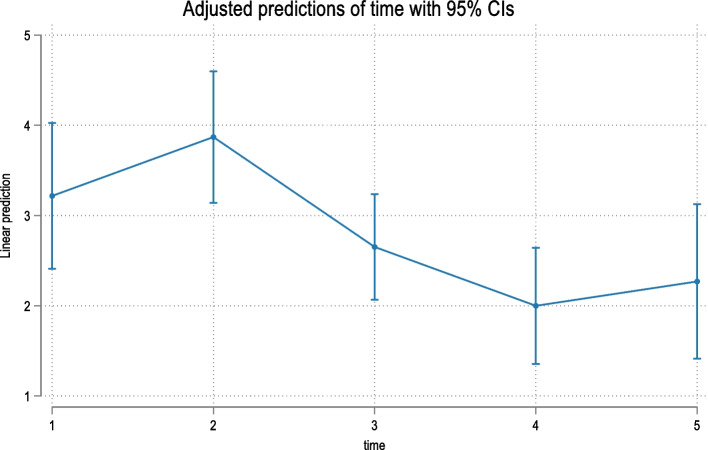
Predicted scores for Q1 over time. T1 = treatment initiation, T2 = one day in treatment, T3 = seven days in treatment, T4 = day 30 in treatment, and T5 = day 30 in the retention phase

**Fig. 2:**
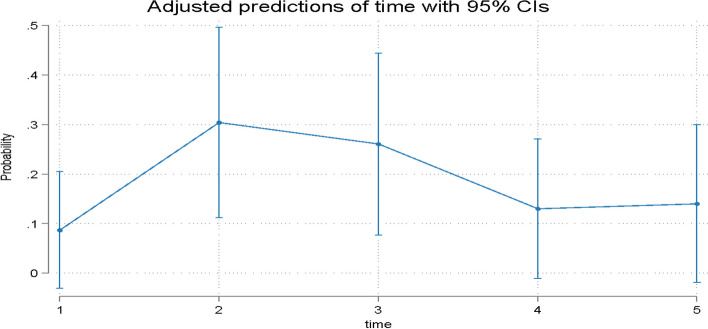
Predicted probabilities of taking medication (Q2) over time. T1 = treatment initiation, T2 = one day in treatment, T3 = seven days in treatment, T4 = day 30 in treatment, and T5 = day 30 in the retention phase

**Fig. 3:**
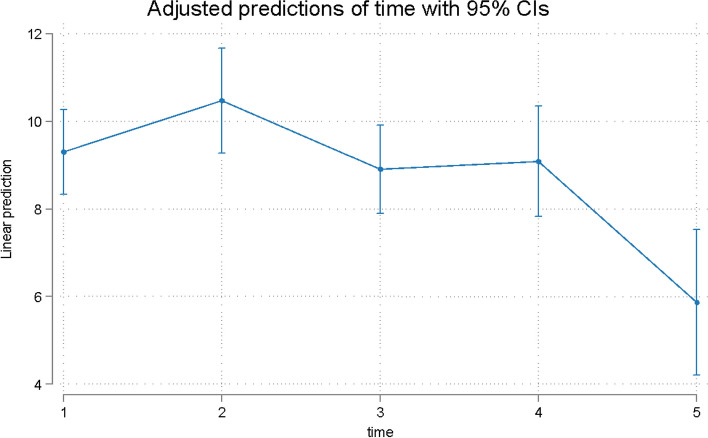
Predicted scores for oral dysfunction in Q3-8 patients over time. T1 = treatment initiation, T2 = one day in treatment, T3 = seven days in treatment, T4 = day 30 in treatment, and T5 = day 30 in the retention phase

**Fig. 4:**
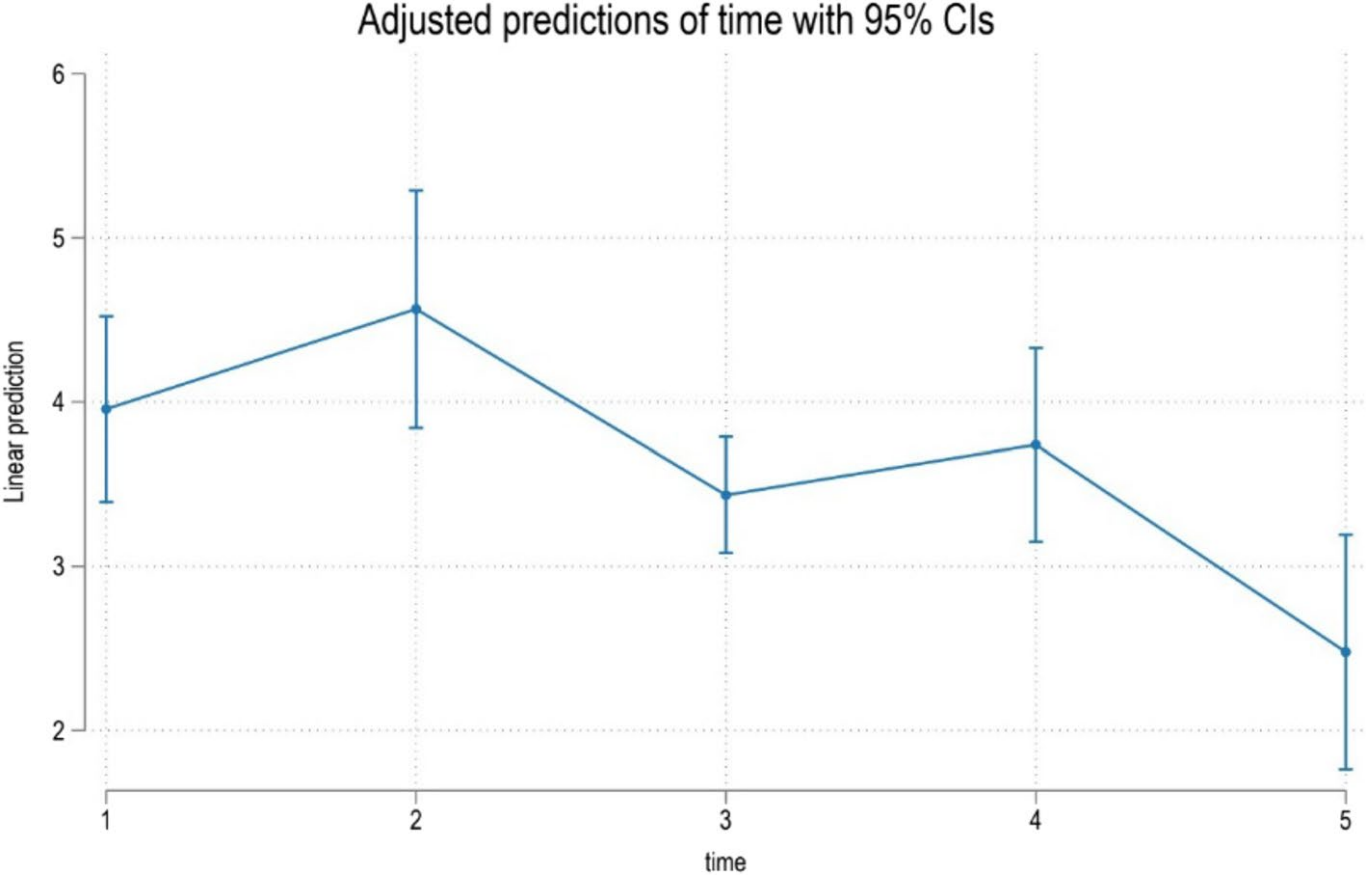
Predicted scores for general activity disturbance in Q9-11 over time. T1 = treatment initiation, T2 = one day in treatment, T3 = seven days in treatment, T4 = day 30 in treatment, and T5 = day 30 in the retention phase

**Fig. 5:**
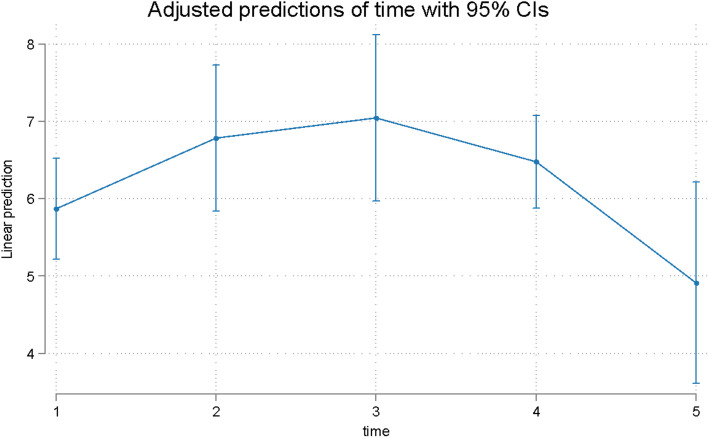
Predicted scores for “not at all” responses in Q12-16 over time. T1 = treatment initiation, T2 = one day in treatment, T3 = seven days in treatment, T4 = day 30 in treatment, and T5 = day 30 in the retention phase

**Fig. 6:**
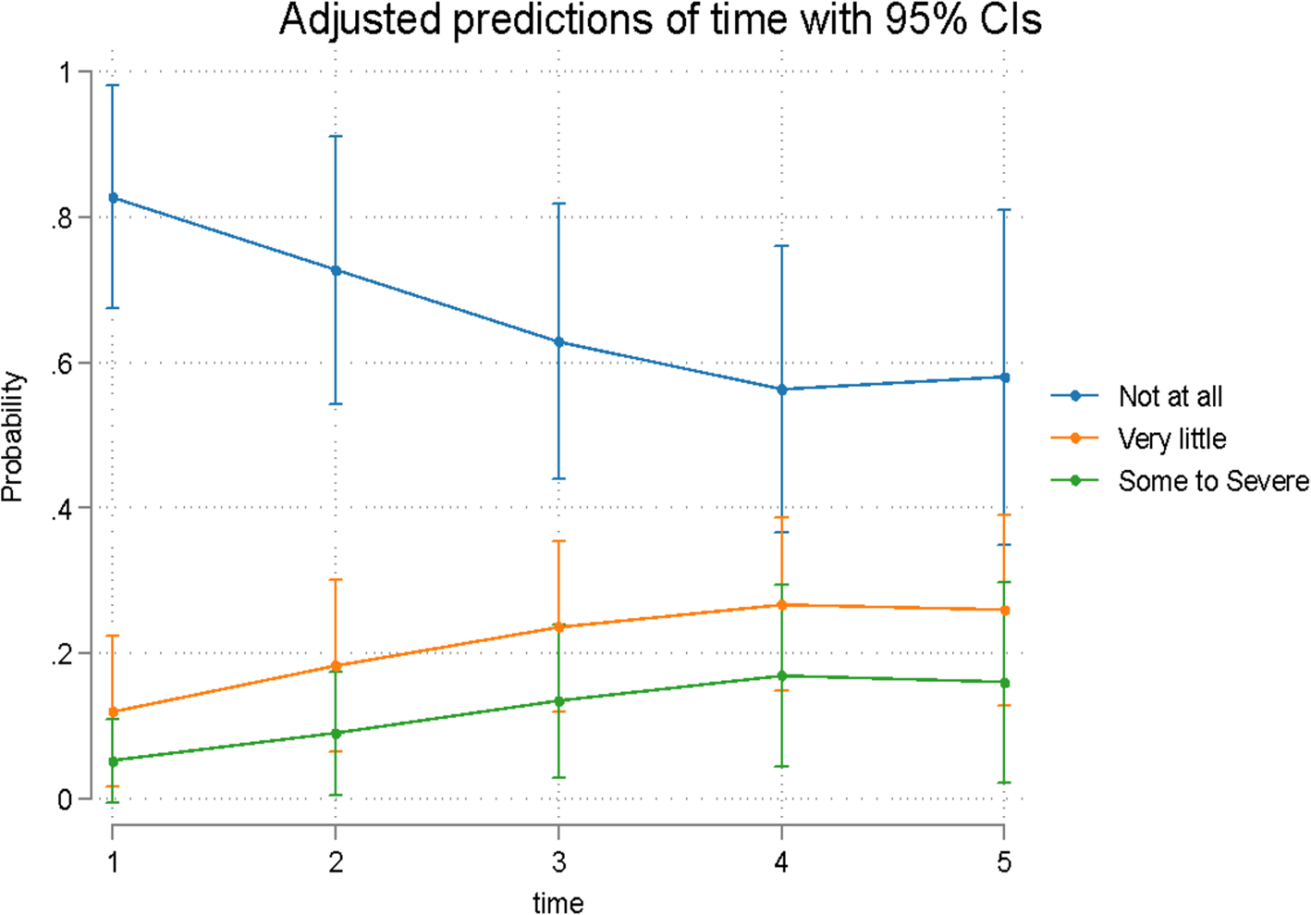
Predicted probabilities for Q17 responses over time. T1 = treatment initiation, T2 = one day in treatment, T3 = seven days in treatment, T4 = day 30 in treatment, and T5 = day 30 in the retention phase

Overall time was a significant predictor (*p* = 0.004) of the response to Question 1 (Q1). An initial increase in the Q1 score was observed at T2, with a decrease over time (Table 2, Fig. 1). Overall time was a significant predictor (*p* = 0.001) of the Q2 response (Table 2, Fig. 2). The remaining questions were assigned to 3 domains in accordance with published evidence [18]. Q3-8 were combined since they evaluate difficulties speaking, swallowing, opening the mouth and eating (oral dysfunction). Overall time was a significant predictor (*p* = 0.001) of the Q3-8 response (Table 2, Fig. 3). Q9-11 assess general activity disturbance by focusing on school/work attendance, sleeping and the ability to participate in routine daily activities. Overall time was a significant predictor (*p* = 0.001) of the Q9-11 response (Table 2, Fig. 4). Q12-16 evaluate other oral symptoms, including bad taste/smell, sores on the cheeks, tongue or lip and food accumulation in the mouth. Overall time had a borderline significant difference (*p* = 0.05) for patients with a Q12-16 response (Table 3, Fig. 5). Q17 evaluates tooth color changes. Its response had 5 levels, but the last 3 levels were merged because of the very low number of events. Overall, the effect of time was not significant (*p* = 0.40) (Table 4, Fig. 6).

**Table 3:** Estimates (95% confidence intervals) and p values for the effect of time on Q1, Q3-8, Q9-11 and Q12-16

	**Pain (Q1)**		**Oral dysfunction (Q3-8)**		**General activity disturbance (Q9-11)**		**Other oral symptoms (Q12-16)**	
Time	Coef. (95% Confidence Interval)	*p* value	Coef. (95% Confidence Interval)	*p* value	Coef. (95% Confidence Interval)	*p* value	Coef. (95% Confidence Interval)	*p* value
T1	reference	0.004*	reference	0.001*	reference	0.001*	reference	0.05*
T2	0.65 (-0.44 to 1.74)	0.24	1.17 (-0.32 to 2.67)	0.12	0.61 (-0.29 to 1.51)	0.18	0.91 (-0.25 to 2.07)	0.12
T3	-0.56 (-1.54 to 0.41)	0.26	-0.39 (-1.81 to 1.03)	0.59	-0.52 (-1.18 to 0.13)	0.12	1.17 (-0.14 to 2.49)	0.08
T4	-1.21 (-2.31 to -0.12)	0.03	-0.22 (-1.86 to 1.42)	0.80	-0.21 (-1.01 to 0.58)	0.59	0.61 (-0.29 to 1.50)	0.19
T5	-0.95 (-2.15 to 0.25)	0.12	-3.43 (-5.44 to -1.43)	0.001	-1.48 (-2.42 to -0.52)	> 0.01	0.96(-2.43 to 0.52)	0.20

^*^Overall *p* value (Wald test)

**Table 4:** Odds ratios (95% confidence intervals) and *p* values for the effect of time on Q1, Q2 and Q17

	**Analgesic intake (Q2)**		**Tooth discoloration (Q17)**	
Time	Odds Ratio (95% Confidence Interval)	*p* value	Odds Ratio (95% Confidence Interval)	*p* value
T1	reference	0.001*	reference	0.40
T2	4.59 (1.22 to 17.36)	0.03	1.80 (0.63 to 5.16)	0.27
T3	3.71 (0.73 to 18.76)	0.11	2.83 (0.93 to 8.67)	0.07
T4	1.57 (0.64 to 3.87)	0.32	3.73 (0.87 to 15.93)	0.08
T5	1.72 (0.23 to 12.58)	0.59	3.48 (0.86 to 14.12)	0.08

^*^Overall *p* value (Wald test)

### STAI questionnaires

**Fig. 7:**
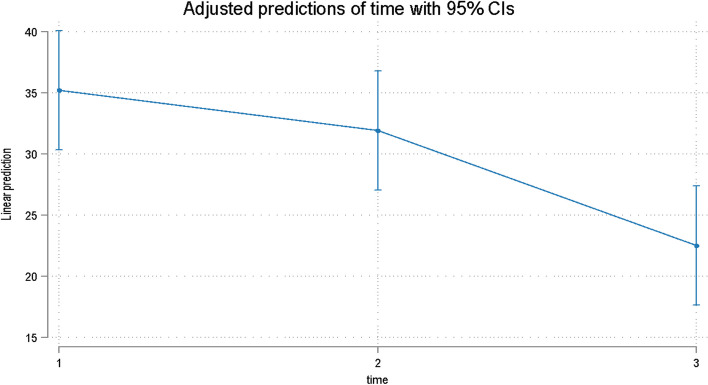
Predicted scores for the STAI Y1 over time

**Fig. 8:**
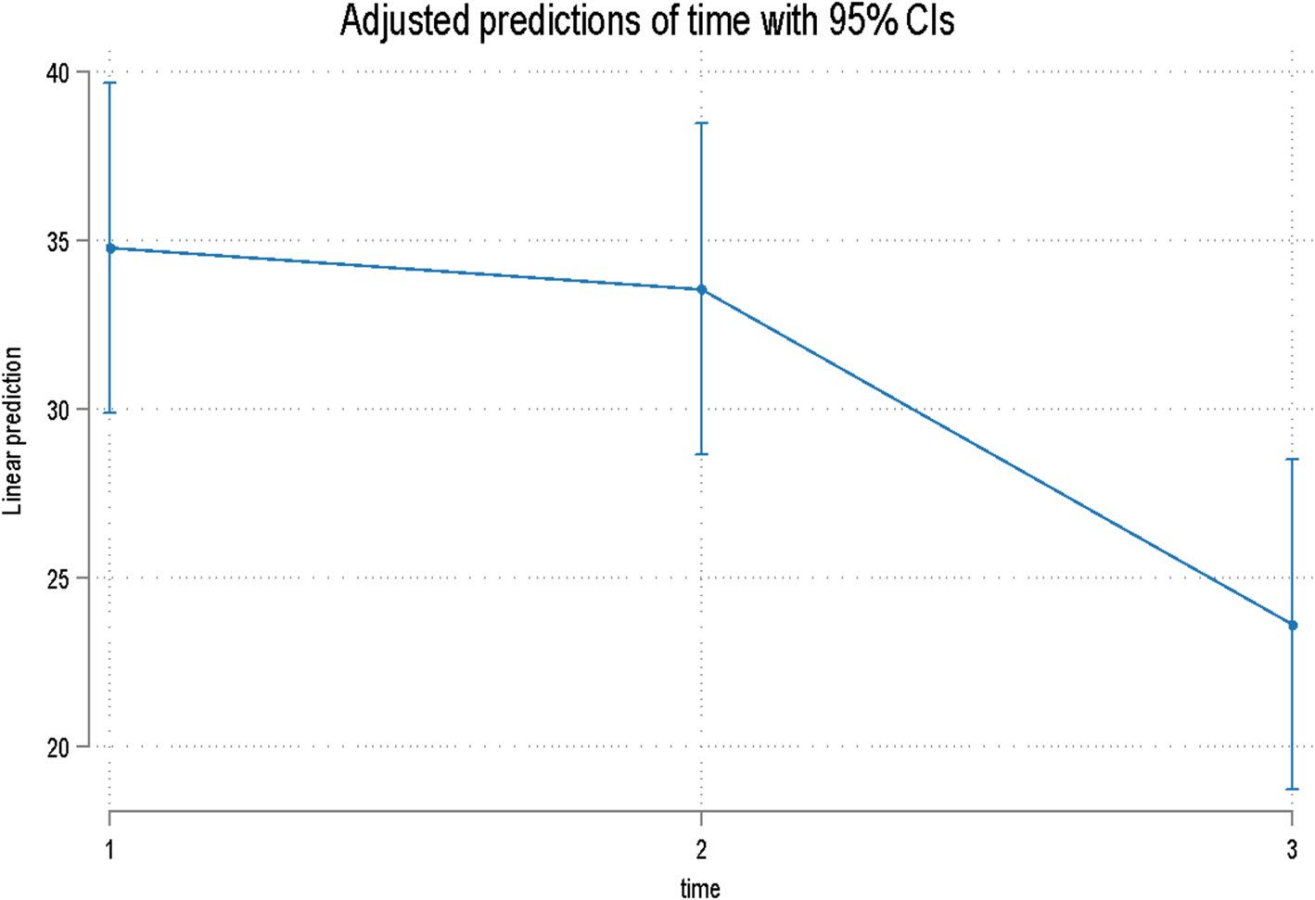
Predicted scores for the STAI Y2 score over time

All patients reported higher depression and anxiety scores during their initial appointment on both the STAI-Y1 and Y2 subscales. Overall time was significant for both the STAI Y1 (Table 5, Fig. 7) and Y2 (Table 5, Fig. 8).

**Table 5:** Estimates (95% confidence intervals) and *p* values for the effect of time on the STAI Y1 and Y2 scores

	**STAI Y1**		**STAI Y2**	
Time	Coef. (95% Confidence Interval)	*p* value	Coef. (95% Confidence Interval)	*p* value
T1	reference	< 0.001*	reference	< 0.01*
T2	-3.30 (-9.81 to 3.20)	0.32	-1.22 (-7.92 to 5.49)	0.722
T3	-12.7 (-19.20 to -6.19)	< 0.001	-11.17 (-17.88 to -4.47)	0.001

^*^Overall *p* value (Wald test)

## Discussion

The increased OHRQoL and STAI anxiety levels experienced by patients over the first days of CAT improved after 1 month of treatment. Recent evidence suggests that in younger patients (children and adolescents), the overall OHRQoL, as well as emotional and social status, improves after orthodontic treatment [21, 39]. Oral health-related quality of life, oral health impact and personality characteristics have been linked to several oral conditions as well as to dental and orthodontic treatments [40–42]. However, these findings have not been consistent with the findings of some studies that failed to prove any such connection [43].

The relationships between personality traits and pain perception and attitude toward orthodontic treatment seem to be significant factors influencing patients’ orthodontic treatment expectations [44]. Compared with males, females report increased pain frequency, duration and severity [45]. Late adolescent and adult patients were chosen for the present study because they represent the majority of patients currently receiving CAT worldwide [46]. Additionally, possible bias due to noncompliance is reduced in adults [23].

### Pain/medication

Even though pain is not always a reliable indication of a pathological situation, patient self-reported pain questionnaires are considered diagnostic tools in both dentistry and orthodontics [47]. The present study demonstrated significantly greater pain levels after 24 h of aligner use. However, these levels decreased at later treatment stages. The peak of medication use occurred the day after treatment initiation, and the medication use decreased at later treatment stages. These findings agree with most of the previous research. The majority (90–95%) of orthodontic patients experience pain during the first weeks of treatment [1, 48], especially after 24 h of appliance placement [23, 49]. During orthodontic treatment, pain and fear are considered the most common patient complaints, often leading to early treatment discontinuation [50–52]. Pain levels may be influenced by age, sex, psychological status, anxiety levels, socioeconomic background, individual pain perception, previous painful experiences and magnitude of orthodontic force [53, 54]. Pain and anxiety are closely related: higher anxiety scores are associated with higher pain levels [55]. In some cases, people may be encouraged by their families at an early age to express their emotions more freely [53]. A review of the dental literature revealed a lack of consensus on the association between pain tolerance and patient age. The pain threshold tends to increase with age [56, 57]. In contrast, conflicting results were obtained by Ngan et al., who failed to associate pain variability with age [49].

Recent studies have shown an association between pain discomfort or analgesic intake and the type/prescription of orthodontic appliances (fixed labial or lingual appliances, CAT) or the prescription of fixed appliances (tip, torque) [23, 58–60]. Compared with those in patients treated with fixed appliances, pain in patients was reportedly lower during the initial days of CAT treatment [26]. Moreover, patients who underwent CAT reported pressure-like pain, whereas patients who used conventional fixed appliances reported more throbbing and duller pain [61]. Nevertheless, a recent study concluded that patient-reported pain during orthodontic treatment may be unpredictable 7 days after initial activation [49]. When patients are fully informed of any possible pain implications during orthodontic treatment, lower pain levels and analgesic medication use are expected during treatment [62, 63].

### Oral function

The patients in the present study reported significantly greater oral function disturbances after 24 h of aligner use. However, these symptoms resolved at later treatment stages. Speech-related problems caused by CAT seemed to be more intense during the first days. Most patients recover to their normal state within 7-14 days, whereas some patients need up to 30-60 days to recover [64]. Both CAT and fixed orthodontic appliances may induce speech problems; however, CAT may affect speech to a greater degree [23, 65].

Food intake and mastication difficulties have been reported in patients undergoing orthodontic treatment [66, 67]. CAT devices have been found to be more comfortable than fixed orthodontic devices [61, 68]. This may be attributed to the fact that CAT patients are instructed to remove their aligners before any chewing activity.

### General activity

The current study demonstrated an increase in patient impairment in terms of their everyday activities, sleep and work during the first day of aligner use. However, these levels decreased at later treatment stages and at the retention phase. A recent trial demonstrated that these sleeping disturbances during CAT or treatment with a lingual appliance may persist for up to 3 months [18]. Patients tend to report higher discomfort levels during the evening and night [1]. However, some studies on patients undergoing CAT did not report any difficulties related to everyday activities, such as social interactions, work, or school performance [58, 61].

### Other symptoms (bad taste/smell, sores, food accumulation in the mouth)

Other symptoms persisted throughout the first week and descended toward the retention phase. A seven-day period seems to give patients the ability to assess any such symptoms, as they have already gained experience with aligners and move on to their second aligner set [69]. However, a recent study concluded that food packing between teeth, affecting 24% of the sample, and pain, affecting 16%, were the most common sources of dissatisfaction immediately after CAT [70].

Patients choosing CAT often seek less irritating and more aesthetic orthodontic devices than traditional buccal fixed appliances [71]. In fact, 70% of patients after 3 months of CAT reported no irritation of the lingual or buccal mucosa [72]. However, compared with lingual appliance-treated patients, aligner-treated patients reported lower scores for tongue sores but higher lip sores scores on the first day after treatment initiation [23]. A recent study reported that patients undergoing CAT may subjectively self-report bad breath and dry mouth symptoms at a high rate in the first three months of orthodontic treatment; however, these symptoms were not confirmed with objective measurements [73].

### Tooth discoloration

A significant number of patients in the present study complained about discoloration at later treatment stages and in the retention phase; however, these findings did not reach statistical significance. The cause of this discoloration is multifactorial. CAT aligners present an ongoing alteration in their surface roughness caused by contact with composite attachments [74], which results in increased hardness depending on the composite filler [75]. Additionally, intraoral aging of aligners may alter their mechanical properties; thus, changes may occur after one week [76]. Moreover, intraoral aligner use may induce composite attachment cracks or fractures [75], which could lead to attachment discoloration. Intraoral aligner exposure to staining solutions may cause material discoloration in various drinks, such as coffee, tea and red wine [76].

### STAI questionnaires

Overall time was a significant predictor of both STAI subscales. Previous studies assessing patient psychological status with these indices reported similar scores during the first month of orthodontic treatment with conventional fixed appliances; these scores were greater on the first day of treatment and decreased over the first month of treatment [77, 78]. It is important to keep patients well informed at the initial treatment stages to reduce anxiety [79].

### Clinical significance of the study

The present study supports clinicians in how to address patients’ complaints during CAT. Patients tend to report higher depression and anxiety levels at the initial CAT stages, even in the absence of attachments and/or IPR. These levels tend to increase over the course of treatment, resulting in a decrease in anxiety scores in the retention phase when no active tooth movements are performed. Patients should be informed that some discomfort and/or pain are expected during the initial stage of treatment; thus, pain killers might be wise to prescribe in advance. The change in tooth color may be attributed to the shading effect that clear aligners induce during intraoral use, and patients must be informed about this phenomenon accordingly. Clinicians should be aware of these difficulties, provide instruction in advance and reassure patients that their initial symptoms tend to improve over time.

### Limitations

The sample in the present study was rather small since the sample size of the aligner treatment arms in most published papers was 30 or more. However, we adopted a robust methodology; i.e., our sample was homogeneous with respect to age and sex and met strict inclusion and exclusion criteria. All patients were treated by the same clinician and with the same aligner system under the same attachment bonding clinical protocol and were given specific instructions concerning aligner maintenance/wear as well as postoperative clinical complications such as pain. Moreover, the protocol did not consider different biomechanical parameters or aligner characteristics, such as precision cuts, pressure points, power ridges, or elastics. The patients presented with little or mild need for orthodontic treatment; however, the association between patient severity and OHRQoL/STAI was not evaluated. The questionnaire used in the present study was used extensively in several previous studies [16–18, 23]; however, questions about eating or food debris or swallowing while wearing appliances are more relevant for patients with fixed appliances.

### Generalizability

The results of the present study may not necessarily be generalizable to other populations.

## Conclusions

- Pain levels and the probability of consuming pain killers were increased one day after initiation of CAT and decreased thereafter.
- OHRQoL scores were negatively affected at the initial phase of the CAT. All the parameters of the index improved at later treatment stages.
- Subjective tooth discoloration did not differ between time points.
- Patients reported higher depression and anxiety scores at the initial appointment, while lower scores were recorded after one month of aligner use and even lower after one month in the retention phase.

## Data Availability

No datasets were generated or analysed during the current study.

## Appendix

The Greek version of the OHRQoL questionnaire [16, 79] was used:

It is important for us to know how orthodontic appliances have affected daily life to improve the quality of care. Please choose the number that corresponds to your assessment over the past 24 h.

1. Rate the worst pain you have felt during the past 24 h on a scale of 1 to 10 (1- not at all, 10 — very much).

2. Have you taken any medication to relieve pain today? (0 = no, 1 = yes).

For the following questions, please use this rating: 1 = no instances, 2 = few instances, 3 = some instances, 4 = several instances, 5 = numerous instances.

3. Has it been difficult to speak today?

4. Has it been difficult to swallow today?

5. Has it been difficult to open your mouth today?

6.Were there any foods you could not eat today?

7. Have you enjoyed your food today?

8. Have you noticed a change in your sense of taste today?

9.Was it difficult to sleep last night?

10. Does the appliance disturb you at work or when studying today?

11. Has it been difficult to continue your daily activities today?

12. Do you have sores on your tongue?

13. Do you have sores on your cheeks?

14. Do you have sores on your lip?

15. Have you had a bad taste or bad smell in your mouth today?

16. Has there been any food debris under the appliance today?

17. Have you noticed a change in the color of your teeth?

Please report any complaints you may have.
